# Structural, Magnetic, and Dielectric Properties of Laser-Ablated CoFe_2_O_4_/BaTiO_3_ Bilayers Deposited over Highly Doped Si(100)

**DOI:** 10.3390/ma17235707

**Published:** 2024-11-22

**Authors:** João Oliveira, Bruna M. Silva, Tiago Rebelo, Pedro V. Rodrigues, Rosa M. F. Baptista, Manuel J. L. F. Rodrigues, Michael Belsley, Neenu Lekshmi, João P. Araújo, Jorge A. Mendes, Francis Leonard Deepak, Bernardo G. Almeida

**Affiliations:** 1 Center of Physics of Minho and Porto Universities (CF-UM-UP), Laboratory for Materials and Emergent Technologies (LaPMET), Departamento de Física, Universidade do Minho, Campus de Gualtar, 4710-057 Braga, Portugal; brunasilva@fisica.uminho.pt (B.M.S.); rosa_batista@fisica.uminho.pt (R.M.F.B.); belsley@fisica.uminho.pt (M.B.); jrm@isep.ipp.pt (J.A.M.); 2 Nanostructured Materials Group, International Iberian Nanotechnology Laboratory (INL), Avenida Mestre José Veiga, 4715-330 Braga, Portugal; leonard.francis@inl.int; 3 Electron Microscopy for Materials Science (EMAT), University of Antwerp, Campus Groenenborger, Groenenborgerlaan 171, 2020 Antwerpen, Belgium; 4 Institute for Polymers and Composites (IPC), Polymer Engineering Department, University of Minho, Campus de Azurém, 4804-533 Guimarães, Portugal; pedro.rodrigues@dep.uminho.pt; 5 Integrated Micro and Nanotechnologies (IMiNa) Group, International Iberian Nanotechnology Laboratory (INL), Avenida Mestre José Veiga, 4715-330 Braga, Portugal; 6 Institute of Physics of Advanced Materials, Nanotechnology and Photonics (IFIMUP), Department of Physics and Astronomy, Faculty of Sciences, University of Porto, Rua Campo Alegre, 4169-007 Porto, Portugal; neenulekshmi@fc.up.pt (N.L.); jearaujo@fc.up.pt (J.P.A.); 7 Instituto Superior de Engenharia do Porto, Instituto Politécnico do Porto, Rua Dr. António Bernardino de Almeida, 4249-015 Porto, Portugal

**Keywords:** barium titanate, cobalt ferrite, bilayer composites, multiferroic, laser ablation, magnetic properties, dielectric properties

## Abstract

Laser ablation was used to successfully fabricate multiferroic bilayer thin films, composed of BaTiO_3_ (BTO) and CoFe_2_O_4_ (CFO), on highly doped (100) Si substrates. This study investigates the influence of BaTiO_3_ layer thickness (50–220 nm) on the films’ structural, magnetic, and dielectric properties. The dense, polycrystalline films exhibited a tetragonal BaTiO_3_ phase and a cubic spinel CoFe_2_O_4_ layer. Structural analysis revealed compression of the CoFe_2_O_4_ unit cell along the growth direction, while the BaTiO_3_ layer showed a tetragonal distortion, more pronounced in thinner BTO layers. These strain effects, attributed to the mechanical interaction between both layers, induced strain-dependent wasp-waisted behavior in the films’ magnetic hysteresis cycles. The strain effects gradually relaxed with increasing BaTiO_3_ thickness. Raman spectroscopy and second harmonic generation studies confirmed BTO’s non-centrosymmetric ferroelectric structure at room temperature. The displayed dielectric permittivity dispersion was modeled using the Havriliak–Negami function combined with a conductivity term. This analysis yielded relaxation times, DC conductivities, and activation energies. The observed BTO relaxation time behavior, indicative of small-polaron transport, changed significantly at the BTO ferroelectric Curie temperature (Tc), presenting activation energies E_τ_ in the 0.1–0.3 eV range for T < Tc and E_τ_ > 0.3 eV for T > Tc. The BTO thickness-dependent Tc behavior exhibited critical exponents ν ~ 0.82 consistent with the 3D random Ising universality class, suggesting local disorder and inhomogeneities in the films. This was attributed to the composite structure of BTO grains, comprising an inner bulk-like structure, a gradient strained layer, and a disordered surface layer. DC conductivity analysis indicated that CoFe_2_O_4_ conduction primarily occurred through hopping in octahedral sites. These findings provide crucial insights into the dynamic dielectric behavior of multiferroic bilayer thin films at the nanoscale, enhancing their potential for application in emerging Si electronics-compatible magneto-electric technologies.

## 1. Introduction

The magnetoelectric (ME) response, in multiferroic systems, is the appearance of an electric polarization upon applying a magnetic field H (direct ME effect) and/or the appearance of a magnetization M upon applying an electric field (converse ME effect) [1,2,3]. In multiferroic magnetoelectric composites made by combining ferroelectric (piezoelectric) and ferromagnetic (magnetostrictive) substances, the magnetoelectric coupling results from the cross-elastic interaction between the two phases, giving a remarkable ME behavior (several orders of magnitude higher than that in the single-phase ME materials so far available) [1,2].

In these composites, an applied magnetic field causes the magnetic phase to deform magnetostrictively, transferring strain to the piezoelectric phase and resulting in electric polarization. The converse effect occurs through a similar mechanism. These static effects have shown promise in recently developed prototype devices for information storage, sensing, actuator applications, and low-power, energy-efficient electronics [3,4,5].

Nanostructured thin-film piezoelectric–magnetostrictive composites have been produced using various methods, including laser ablation, sputtering, and sol–gel techniques [4,6]. Laser ablation stands out for its ability to control deposition at the atomic level, minimizing extrinsic effects. Notably, laser-ablated thin films have demonstrated high magnetoelectric coupling values, reaching up to ~20.75 V·cm^−1^·Oe^−1^ in the multilayered BaTiO_3_/BiFeO_3_ system [7,8].

While static multiferroic behavior has been extensively studied, the dynamic frequency-dependent properties of multiferroic composite thin films remain relatively unexplored [9,10,11,12]. The impact of strain on frequency-dependent electric and magnetic behaviors, particularly the role of strain-induced resonances in enhancing coupled magnetic and electric interactions at the nanoscale, requires further investigation [12]. An improved understanding of dynamic multiferroic behavior can further enable the development of fast spintronic devices with enhanced responses. Additionally, the influence of thin-film layer thicknesses on these properties needs clarification.

Frequency-dependent studies, such as impedance spectroscopy, are crucial for addressing these aspects [9,10,13]. Temperature-dependent impedance spectroscopy, which examines complex electric and dielectric properties, allows for the deconvolution and characterization of contributions to the dielectric and resistive properties of low-dimensional nanostructures [9]. This technique enables the distinction between extrinsic effects (e.g., defects, grain boundaries, conductivity) and intrinsic responses, providing valuable insights into the physical mechanisms governing interlayer interactions within composite multiferroic nanostructures.

Multiferroic composites based on BaTiO_3_ (piezoelectric) and CoFe_2_O_4_ (magnetostrictive) have been produced in both bulk [8,14,15] and as thin films [8,16,17]. However, studies of their dielectric and impedance behavior have primarily focused on bulk materials, leaving their dynamic electric and dielectric behavior at the nanoscale in nanostructured bilayer or multilayer thin films largely unexplored. Furthermore, their deposition on silicon substrates is favorable for integration with current silicon electronics. In this context, current thin-film ferroelectric and multiferroic systems are commonly deposited on perovskite oxides with similar unit-cell dimensions, such as SrTiO_3_ (an insulator), Nb-doped SrTiO_3_ (which offers high conductivity, serving as a bottom electrode), or SrRuO_3_ (also for bottom electrode) [5]. Other oxide single-crystal substrates have also been employed, including LaAlO_3_, (LaAlO_3_)_0.3_-(SrAl_0.5_Ta_0.5_O_3_)_0.7_, ReScO_3_ (Re = Dy, Gd, Sm, and Nd), MgO, Al_2_O_3_, and TiO_2_ [5], as they can modify the strain state of the film through interfacial lattice mismatch. However, these oxide substrates pose limitations for applications due to their high cost, small size, complex deposition process, and lack of compatibility with current silicon technology. Beyond oxides, Si/SiO_2_/Ti/Pt(100) substrates with a conductive Pt top layer have also been used [2], although they are expensive and complex to fabricate. Consequently, the potential for depositing films directly on Si wafers has gained considerable attention [5]. In this context, the direct fabrication of BTO\CFO bilayer magnetoelectric nanostructures on highly doped conductive Si substrates enables compatibility with current CMOS technology, widening the prospects for applications.

BaTiO_3_ is a ferroelectric perovskite with promising applications in high-performance, lead-free piezoelectric devices [18,19]. At elevated temperatures, it adopts a cubic structure, where 12 neighboring oxygen atoms surround the large barium ions, and 6 oxygen atoms surround each titanium ion in an octahedral arrangement. BaTiO_3_ undergoes several phase transitions with temperature, including a transition from the high temperature cubic phase to a tetragonal structure at 130 °C [20,21]. As the temperature drops further, it transitions to an orthorhombic phase at around 5 °C and finally to a rhombohedral phase near −90 °C [19]. The tetragonal phase at room temperature is ferroelectric, while the cubic phase at higher temperatures is paraelectric [19,20,21].

Cobalt ferrite (CoFe_2_O_4_) is a ferrimagnetic material with a cubic inverse spinel structure [22]. Its magnetic properties arise from complex interactions between Fe^3+^ and Co^2+^ ions occupying tetrahedral and octahedral sites. Spins of ions in octahedral sites interact ferromagnetically, while Fe^3+^ ions in tetrahedral sites align antiferromagnetically with those in octahedral sites due to superexchange interactions mediated by O^2−^ ions [23]. This arrangement partially cancels Fe^3+^ magnetic moments, resulting in a net magnetic moment primarily from Co^2+^ ions. The high magnetocrystalline anisotropy of CoFe_2_O_4_, its significant magnetostriction, and excellent mechanical stability make it an ideal candidate for magnetoelectric composite thin films [1,6,23].

In this study, thin bilayer composite films consisting of a BaTiO_3_ (BTO) layer deposited over a CoFe_2_O_4_ (CFO) film have been prepared on (100) oriented highly As-doped conductive Si substrates, compatible with current Si electronics. The BTO layer thickness was varied, and the CFO layer thickness was kept approximately constant. The films’ structural, microstructural, spectroscopic, non-linear optical, magnetic, and dielectric properties were characterized. Their analysis revealed the ferroelectric tetragonal structure of the BTO layer and the cubic spinel structure of CFO. Magnetic hysteresis loops exhibited strain-induced wasp-waisted behavior due to BTO-CFO interfacial mechanical interaction, which decreased with increasing BTO thickness. The electrical permittivity of the bilayer films was modeled and fitted to obtain characteristic temperatures, activation energies, and relaxation behavior. The results provide insights into small-polaron transport in the relaxation behavior of the BTO layer, the influence of the ferroelectric-paraelectric transition, BTO critical exponents, universality class, and CFO conductivity behavior as a function of BTO layer thickness.

## 2. Materials and Methods

Bilayer nanocomposite thin films of barium titanate (BaTiO_3_) deposited over a cobalt ferrite (CoFe_2_O_4_) layer were prepared by pulsed laser ablation on the n-type arsenic-doped conductive Si (100) substrates WAFERS2-NFR (Neyco, Vanves, France), with a resistivity ranging between 0.001 Ω∙cm and 0.005 Ω∙cm. The BTO ceramic target was produced using BaTiO_3_ nanoparticle powder (SIGMA-ALDRICH, Saint Louis, MI, USA), with particle sizes below 100 nm and a purity of at least 99.0%. The CFO ceramic target was produced using CoFe_2_O_4_ nanoparticle powder (Nanostructured and Amorphous Materials, Katy, TX, USA) with particle sizes between 35 nm and 55 nm and a purity of 98%. The powders were placed into a pellet die (PerkinElmer, Waltham, MA, USA) and pressed using a Carver hydraulic press (model 4350 L, Wabash, IN, USA) at a pressure of 220 MPa, forming cylindrical targets with a diameter of 13 mm and a thickness of 3 mm. The targets were then annealed in a Carbolite MTF 12/38/250 tube furnace (Hope, Derbyshire, United Kingdom) at 1000 °C—held for 18 h for BTO and 24 h for CFO. The temperature was ramped up and down at a rate of 2 °C/min.

The depositions were performed with a KrF excimer laser LPX PRO 210 (Lambda Physik—Coherent, Saxonburg, PA, USA) with a wavelength λ of 248 nm at a fluence of 2 J/cm^2^. The samples were produced with varying layer deposition times, while the other preparation conditions were kept constant. The deposition parameters for each layer are summarized in Table 1. They were based on previous results for laser-ablated films of both materials [6,24].

The pressure inside the deposition chamber was initially lowered to 5 × 10^−2^ mBar using a rotary vacuum pump Pascal 2010 I (Alcatel, Annecy, France). The pressure was further reduced to 10^−5^ mBar using the turbomolecular vacuum pump ATP 80 (Adixen—Alcatel, Annecy, France). Pressures above 10^−2^ mBar were measured with a Pirani gauge (PGC1, AML, Arundel, UK), while pressures below 10^−2^ mBar were measured with a cold cathode vacuum gauge, model 943 (MKS, Andover, MA, USA). The substrate temperature was increased to the target temperature at a rate of 10 °C/min using a resistive-heating substrate holder (TSST, Enschede, The Netherlands), with the temperature measured with a thermocouple probe type K (Omega, Norwalk, CT, USA). Before deposition, the substrates were kept at 650 °C for 30 min to promote the evaporation of residuals from the surface of the substrate. After deposition, the samples were cooled down to room temperature at a rate of 10 °C/min, with an oxygen pressure of 1 mbar to prevent under-oxidation of the films [25,26]. Under these conditions, the deposition rates determined from cross-sectional scanning electron microscopy micrographs were 2 nm/min for cobalt ferrite and 10 nm/min for barium titanite. Four bilayer samples were produced under these conditions, with approximately the same CoFe_2_O_4_ thickness and varying deposition times for the BaTiO_3_ layer.

The structural analysis of the samples was conducted using X-ray diffraction (XRD), with a D8 Discover (Bruker, Billerica, MA, USA) diffractometer, using Cu-Kα radiation (λ = 1.54060 Å). Each sample was scanned in the 2θ range from 15° to 60°, with an angular step size of 0.02°. Grazing incidence measurements were performed with a fixed incidence angle of 1° and in the 2θ range of 20° to 50°.

The morphology was characterized by scanning electron microscopy (SEM) with a Quanta 650 FEG (FEI, Hillsboro, OR, USA) with an acceleration voltage of 20 keV. Cross-sectional scanning electron micrographs of the bilayer thin films were obtained at a 45° tilt. Microanalysis to determine the chemical composition of the samples was performed by energy-dispersive X-ray spectroscopy (EDX) using a Flat Quad 5060F (Bruker, United States of America, Billerica) detector at an acceleration voltage of 20 kV. Raman spectroscopy was performed in the range of 50 cm^−1^ to 1000 cm^−1^ with a LabRAM HR Evolution (Horiba, Japan, Kyoto) spectrometer using a diode laser with an excitation wavelength of 532 nm.

Impedance spectroscopy was used to measure the dielectric properties of the samples. In these measurements, the sample was treated as a parallel-plate capacitor included in an LCR network [27]. Circular gold electrodes, with a diameter of approximately 1 mm, were deposited on the surface of the samples by sputtering. The highly conductive Si(100) substrate where the samples were deposited served as the bottom electrode for the bilayer films, while a gold (Au) contact was used as the top electrode. The capacitance was measured with a model 6440A (Wayne Kerr, Bognor Regis, UK) precision component analyzer in the frequency range of 20 Hz to 3 MHz. Three measurements were made on each sample to ensure reproducibility. The complex permittivity, expressed as ε = ε′ − iε″, where ε′ and ε″ are the real and imaginary parts, respectively, was calculated from the measured capacitance (C) and loss tangent (tan⁡δ), using Equations (1) and (2).

(1) $$C=\varepsilon^{\prime }\varepsilon_{0}\frac{A}{d}$$

(2) $$\tan\delta =\frac{\varepsilon^{\prime \prime }}{\varepsilon^{\prime }}$$

where *d* is the thickness of the thin film, *A* is the area of the electrode, and *ε*_0_ is the vacuum permittivity. The temperature-dependent dielectric measurement was conducted between room temperature and 200 °C, with the temperature changing at a rate of 0.5 °C/min, using a PL 706 PID (Polymer Laboratories—Thermal Sciences, Epsom, UK) controller and furnace for temperature control. The temperature was measured using a PT100 resistance model 1PT100FR828 (Omega, Manchester, UK), with the voltage measured by a voltmeter model 182 (Keithley, Cleveland, OH, USA). Shielded test leads were used to prevent parasitic impedances from the connecting cables.

The second-order non-linear optical response of the bilayer films was investigated using a mode-locked Ti:Sapphire laser Mira 900 (Coherent, Saxonburg, PA, USA) that operated with a pulse repetition rate of 76 MHz. This laser system produced pulses with a duration of approximately 120 fs and a central wavelength of 795 nm. The laser output was typically attenuated to around 1.3 nJ per pulse. The measurements were carried out using a custom-built microscope with a Nikon plan-fluorite 10× objective (with an effective focal length of 20 mm and numerical aperture of 0.3). The focused laser spot size was estimated to be approximately 3 microns in diameter, which resulted in incident fundamental fluxes of roughly 30 mJ/cm^2^.

A reflection geometry was employed to measure the second harmonic signal due to the opacity of the silicon substrates. Fixed calcite polarizers and half-wave plates controlled the polarizations of the incident fundamental and detected second harmonic light. A combination of low-pass and band-pass filters effectively reduced the reflected fundamental light. The filtered signals were then focused into a multimode fiber connected to a 0.3 m spectrophotometer Shamrock 303i (Andor—Oxford Instruments, Belfast, UK) to further isolate the second harmonic signal. Finally, a cooled CCD camera Newton 920 (Andor—Oxford Instruments, Belfast, UK) was utilized to capture the isolated second harmonic signals digitally, integrating them over a time window of 60 s.

## 3. Results and Discussion

### 3.1. Structural and Morphological Characterizations

**Figure 1:**
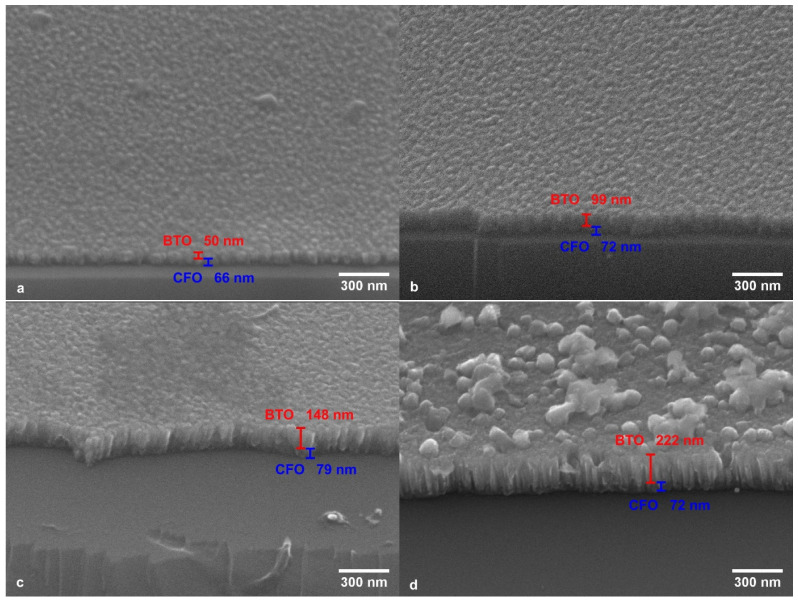
Scanning electron micrographs of the cross-sections of the samples: (**a**) SCB-05, (**b**) SCB-10, (**c**) SCB-15, and (**d**) SCB-22 with the measured thickness of the CoFe_2_O_4_ and BaTiO_3_ layers.

Figure 1 shows scanning electron micrographs of the cross-section of the samples. In these micrographs, columnar growth of the films is observed. The surface morphology of the samples reflects the tops of the columns that make up the films. The layer thicknesses were measured from the cross-section, as shown in Figure 1, and are presented in Table 2. Accordingly, the samples were named following the nomenclature: SCB-thickness, where S is the Si substrate, CB represents the initials of the CFO and BTO layers, and thickness indicates the BTO layer thickness in tens of nanometers, as shown in Figure 1. As expected, the CoFe_2_O_4_ layer thicknesses were similar in the different samples, while the BaTiO_3_ layer thickness increased from 50 nm to 222 nm.

**Figure 2:**
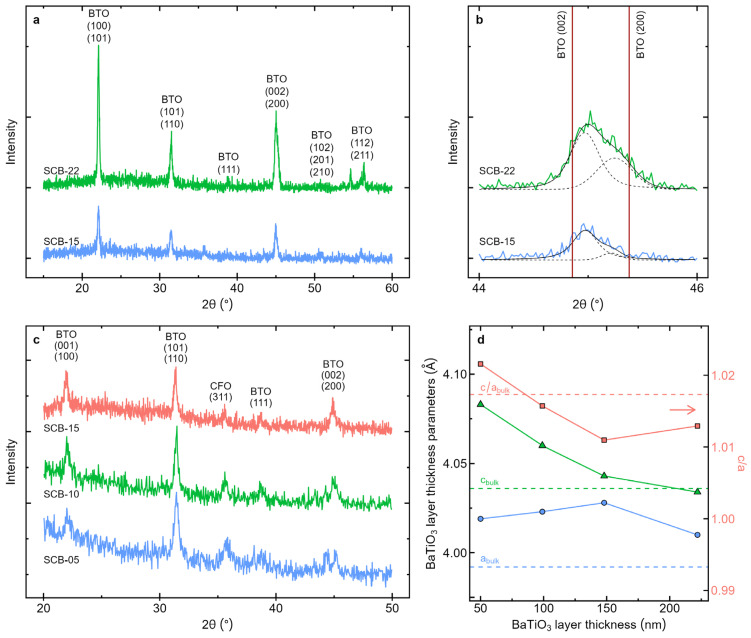
In (**a**), the X-ray diffractograms of the bilayer samples SCB-15 and SCB-22 are shown. In (**b**), an enlargement of (**a**) is displayed, focusing on the 2θ region between 44° and 46°. The vertical lines mark the peak positions of the tetragonal BaTiO_3_ phase, while the black lines represent the pseudo-Voigt fits for the (002)/(200) BaTiO_3_ peaks. In (**c**), the grazing incidence X-ray diffractograms of the bilayer samples SCB-05, SCB-10, and SCB-15 are presented, where the CoFe_2_O_4_ (311) peak is visible. In (**d**), the lattice parameters of the BaTiO_3_ layer (left axis) and the c/a ratio (right axis) are shown. The bulk lattice parameter values, a = 3.992 Å and c = 4.036 Å, were taken from reference [28].

The structure of the samples was studied by X-ray diffraction, and the results are presented in Figure 2. The X-ray diffractograms of the samples with the thickest BaTiO_3_ layer are shown in Figure 2a. The diffractograms display BaTiO_3_ peaks with multiple orientations, indicating that the films are polycrystalline without a preferential growth direction. Figure 2b shows an enlargement of the diffractogram in Figure 2a, focusing on the angular region around the (200) and (002) diffraction lines. The broadening and asymmetry of these peaks indicate the presence of the BaTiO_3_ polar tetragonal phase in the films at room temperature. Figure 2c shows grazing incidence measurements for the three samples with thinner BaTiO_3_ layers. These measurements indicate the presence of the (311) peak of the CoFe_2_O_4_ spinel ferrite phase, with the intensity slightly decreasing as the thickness of the top BaTiO_3_ layer increases.

The X-ray diffraction peaks were fitted using pseudo-Voigt functions to determine their angular positions and full width at half maximum (FWHM), as shown in Figure 2b. From the angular peak positions, the interplanar distances *d*_hkl_ were determined using the Bragg equation. For CFO (cubic), the lattice parameter *a* was determined using the (311) peak and the relation for the cubic system $d_{hkl}=a/\sqrt{h^{2}+k^{2}+l^{1}}$ [29]. For BTO (tetragonal), the *a* and *c* lattice parameters were calculated using the (002) and (200) peaks and applying the relation for a tetragonal system $d_{hkl}=a/\sqrt{h^{2}+k^{2}+l^{1}(a^{2}/c^{2})}$ [29].

The grain sizes of the layers were calculated using the Scherrer equation expressed as L=0.9λ/(Bcos(θ)), where *L* is the grain size, *B* is the full width at half maximum of the peak and *θ* is the peak angle [29]. The equation was applied to the most intense X-ray diffraction peaks: (002)/(200) for the BaTiO_3_ and (311) for the CoFe_2_O_4_. Table 2 displays the CoFe_2_O_4_ lattice parameters and the grain sizes determined from the X-ray diffractograms. The obtained grain sizes tend to increase for higher BaTiO_3_ layer thicknesses. This is due to the longer exposure of the CoFe_2_O_4_ and BaTiO_3_ layers to high temperatures, promoting grain growth due to a higher deposition time. Also, the lattice parameter for the CoFe_2_O_4_ is always below the corresponding bulk value, indicating the presence of contraction strain along the growth direction of the CFO layer in the thin films.

Figure 2d shows the lattice parameters of the BaTiO_3_ layer, as determined from the XRD measurements. The lattice parameter a_BTO_ fluctuates between 4.10 Å and 4.23 Å, which is higher than the bulk value. On the other hand, c_BTO_ is higher for low BaTiO_3_ thicknesses and decreases with increasing BaTiO_3_ layer thickness. In this context, it is well known that ferroelectric nanoscopic films exhibit a lower tetragonality (c/a ratio) compared to their bulk counterparts [20], which is also observed in the c/a ratio of our samples (Figure 2d). However, here, in the case of very thin films, interfacial elastic interactions induce stresses that promote the stabilization of the ferroelectric phase, increasing the tetragonality of the BaTiO_3_ layer at smaller thicknesses. Conversely, as the thickness increases, the interfacial interaction becomes progressively less significant, reducing the tetragonality, as seen in the films. Therefore, the observed increase in c/a for thinner BaTiO_3_ layers indicates a strong mechanical interaction between the CoFe_2_O_4_ and BaTiO_3_ layers in the composite bilayer multiferroic samples.

**Figure 3:**
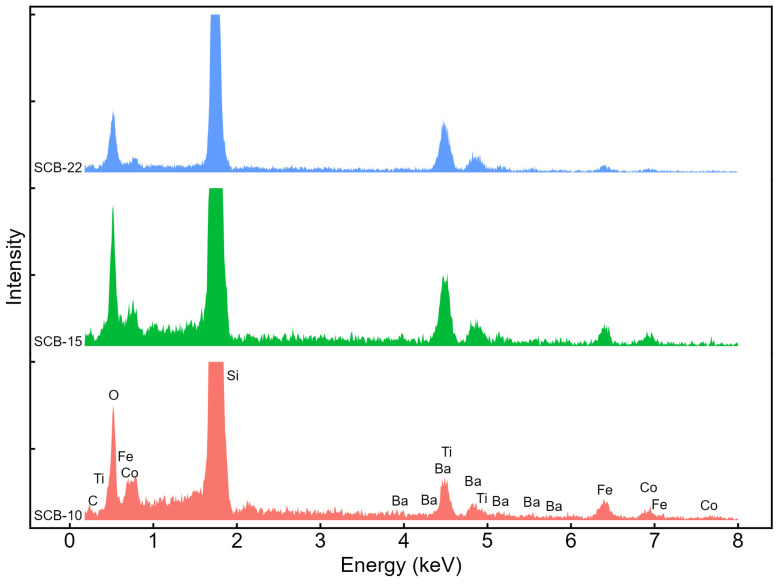
Energy-dispersive X-ray spectrograms of the SCB-10, SCB-15, and SCB-22 samples.

Figure 3 presents the energy-dispersive X-ray spectroscopy (EDX) analysis of the samples, where all the atomic elements of the thin films are detected. The only impurity element observed is carbon, likely due to atmospheric exposure. Table 3 summarizes the atomic percentages determined by the EDX measurements, as well as the Co/Fe and Ba/Ti ratios. The values are approximately as expected for the CFO and BTO layers. The oxygen content values for each layer are not included, as they are influenced by the presence of oxygen in both materials.

### 3.2. Raman Spectroscopy

Bulk barium titanate undergoes a phase transition at around 130 °C [21,30], from a high-temperature cubic (paraelectric) phase to a tetragonal ferroelectric structure, which is retained down to room temperature. In nanoscopic materials, the small grain size composing the films results in broad peaks in X-ray diffraction, making it difficult to distinguish the presence of the tetragonal phase, as it corresponds to only a slight distortion of the cubic phase. However, a broadening of the (200) diffraction peak is observed, with increasing asymmetry towards higher angles (Figure 2), suggesting the stabilization of a polar tetragonal phase of BaTiO_3_.

In this respect, the cubic (paraelectric) phase of BaTiO_3_ has a Pm3m (Oh) crystal symmetry, which, theoretically, lacks Raman-active modes [31,32]. However, distortions in the perfect cubic cell, caused by disorder in the Ti positions or external stresses on the grains, can occur, leading to broad peaks around 250 cm^−1^ and 520 cm^−1^ in polycrystalline powders [32]. On the other hand, the tetragonal phase has P4mm symmetry, with 15 predicted modes: 3[A1(TO) + A1(LO)] + B1 + 4[E(TO) + E(LO)]. Therefore, Raman spectroscopy can detect the presence of the ferroelectric tetragonal phase in the samples through the appearance of these peaks.

**Figure 4:**
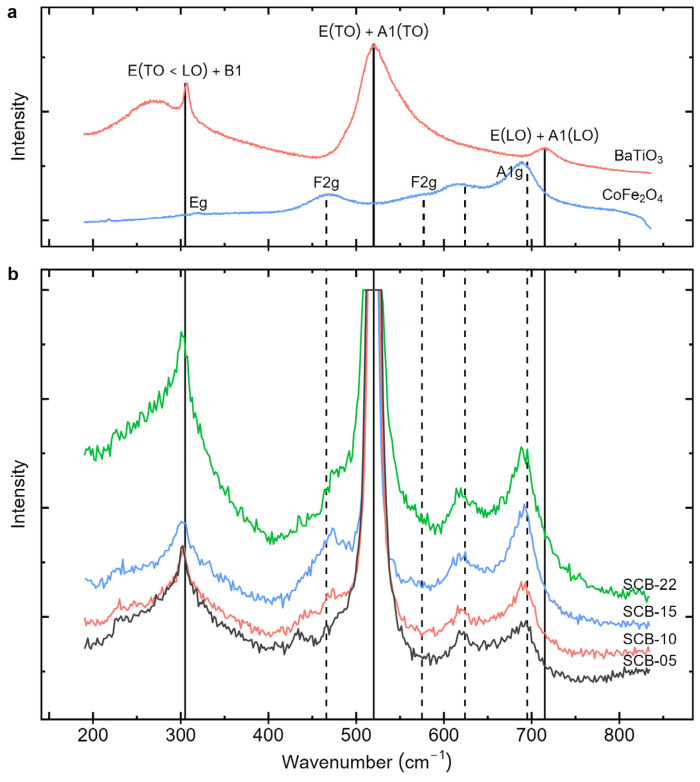
Raman spectra of (**a**) bulk reference powders of CoFe_2_O_4_ and BaTiO_3_, and (**b**) the bilayer thin films of CoFe_2_O_4_/BaTiO_3_. The vertical dashed lines indicate the CoFe_2_O_4_ vibration modes, while the solid vertical lines indicate the BaTiO_3_ modes.

Figure 4 shows the measured Raman spectra of the bilayer samples and those of cobalt ferrite and barium titanate bulk powders used for reference. The band observed around 520 cm^−1^ in all the samples is due to the silicon substrate.

Bulk cobalt ferrite has an inverse spinel structure with the space group $Fd3\bar{m}$. Factor group analysis yields 39 vibrational modes, five of which are Raman-active [33,34]. They are at 210 cm^−1^ (F_2g_), 312 cm^−1^ (E_g_), 466 cm^−1^ (F_2g_), 575 cm^−1^ (F_2g_), and 695 cm^−1^ (A_1g_) [33]. The Raman modes of cobalt ferrite below 600 cm^−1^ originate from symmetric and anti-symmetric bending of oxygen atoms in the M-O bond (M = Co, Fe) in the octahedral sublattice, while the modes above 600 cm^−1^ are due to symmetric stretching of oxygen atoms relative to the metal ions in the tetrahedral sublattice [34]. Three main CoFe_2_O_4_ peaks are observed in the spectra. The peaks above 600 cm^−1^ correspond to the A_1g_ modes related to the symmetric stretching of O-M bonds. Additionally, a band appears near 470 cm^−1^ for low BaTiO_3_ thicknesses, with its intensity decreasing as BaTiO_3_ content increases in the bilayers. This band can be assigned to an F_2g_ mode of CoFe_2_O_4_.

On the other hand, for BaTiO_3_, the band around 305 cm^−1^ tends to intensify with increasing BaTiO_3_ thickness, indicating that it originates from the BTO layer. This band can be associated with the [B1, E(TO + LO)] mode of BaTiO_3_ in the tetragonal phase [31]. The presence of this mode further confirms the stabilization of the BaTiO_3_ tetragonal ferroelectric phase alongside the CoFe_2_O_4_ cubic spinel structure in the bilayer films.

### 3.3. Second Harmonic Generation

**Figure 5:**
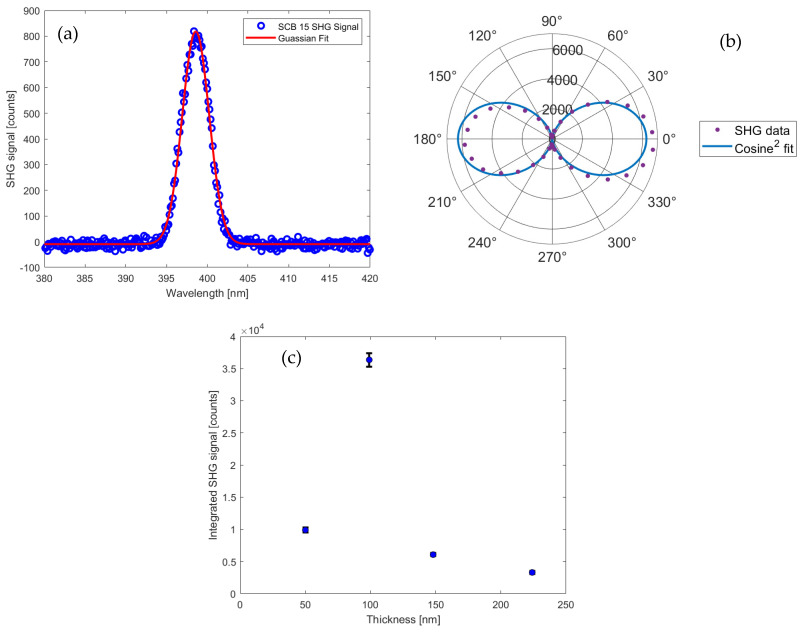
Second harmonic spectra generated by the bilayer samples. (**a**) An example of the raw data together with the associated Gaussian fit for sample SCB 15. (**b**) The polarization dependence of the generated second harmonic light’s integrated signal for sample SBC 15. The blue curve is the best fit cosine-squared dependence. (**c**) The variation of the integrated second harmonic signal along with the BTO films’ estimated thickness. The error bars indicate the 95% confidence interval based on the fitted Gaussian amplitude and widths.

Optical second harmonic generation (SHG) measurements were conducted to further confirm the non-centrosymmetric nature of the BaTiO_3_ phase in the produced bilayers. Figure 5 presents the results of these measurements, demonstrating detectable second harmonic signals from all samples. This observation conclusively verifies the non-centrosymmetric (ferroelectric) structure of the barium titanate phase at room temperature.

The intensity of the SHG signals was quantified by fitting each spectrum to a Gaussian function and integrating the resulting fit. Figure 5a displays a representative spectrum for sample SCB 15, while Figure 5b illustrates the polarization dependence of the generated second harmonic light for the incident polarization that produced the strongest signal. The second harmonic light exhibits strong linear polarization, with a minor discrepancy between the integrated signals at 0 and 180 degrees, potentially due to slight sample degradation during measurements caused by high incident fluxes (approximately 30 mJ/cm^2^).

Figure 5c reveals the relationship between the estimated integrated SHG signal and the BTO layer thickness. Notably, sample SCB 10, with an estimated thickness of 99 nm, generated the most intense second harmonic signal in reflection. This sample also exhibited a lattice parameter ratio (c/a) closest to the bulk value for BTO (refer to Figure 2d).

These results not only confirm the non-centrosymmetric nature of the BTO phase but also suggest the potential for tailoring the second-order non-linear optical properties of these bilayer structures through precise control of layer thickness.

### 3.4. Magnetic Properties

**Figure 6:**
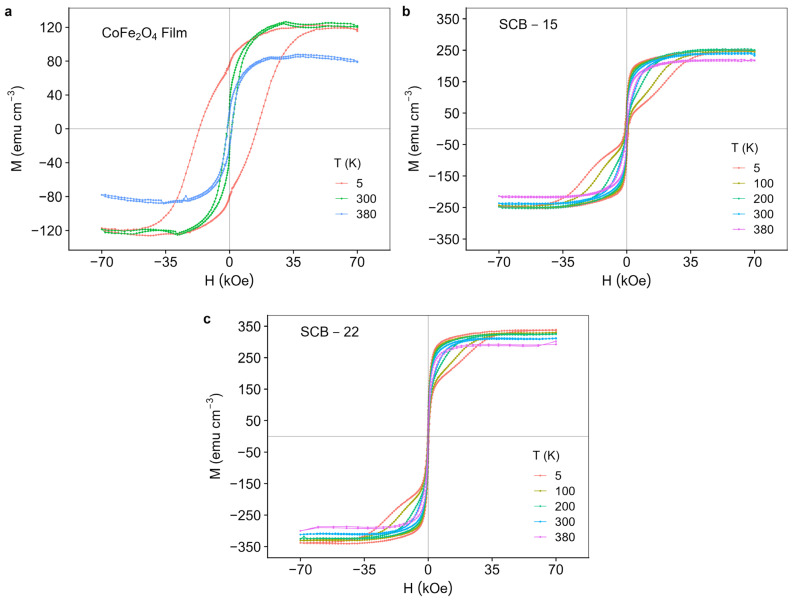
Magnetic hysteresis cycles measured at different temperatures for (**a**) pure CoFe_2_O_4_ thin film and for the bilayer samples (**b**) SCB-15 and (**c**) SCB22.

Figure 6 shows the temperature-dependent magnetic hysteresis cycles measured for the bilayer samples with different BaTiO_3_ thicknesses alongside a pure CoFe_2_O_4_ thin film.

The shape of the loops for the bilayer films exhibits a wasp-waisted behavior [35], which is absent in the single-phase pure CoFe_2_O_4_ film. In this regard, previous studies have highlighted the importance of interfacial strain on the shape of hysteresis loops and the emergence of the wasp-waisted effect [35,36,37,38]. Based on the XRD results, the CoFe_2_O_4_ and BaTiO_3_ layers in samples with thinner BaTiO_3_ are more strained. As the BaTiO_3_ thickness increases, the surface-to-volume ratio decreases, reducing the importance of the mechanical interfacial interaction between the CoFe_2_O_4_ magnetic layer and the BaTiO_3_ film, which results in strain relaxation. Films grown with thinner BaTiO_3_ layers, exhibiting greater strain, show lower hysteresis compared to thicker samples and the pure CoFe_2_O_4_ film. Additionally, they display a well-defined step-like shape at field values around 10 kOe. This step-like feature suggests the presence of two distinct magnetic phases—hard and soft—whose relative contributions depend on thickness. The soft phase is likely related to the interfacial effect between the CoFe_2_O_4_ and BaTiO_3_ layers, as it varies with BaTiO_3_ thickness. Therefore, the contribution of the soft phase decreases as the total thickness increases, making this feature less evident in thicker films [38]. This is accompanied by a decreased wasp-waisted behavior and increased coercive field as the BaTiO_3_ thickness increases due to the decrease in interfacial-induced strain. Thus, the magnetic results underscore the crucial role of mechanical coupling between the cobalt–ferrite phase and the barium–titanate layer in the films, particularly in the thinner samples.

### 3.5. Impedance Spectroscopy

**Figure 7:**
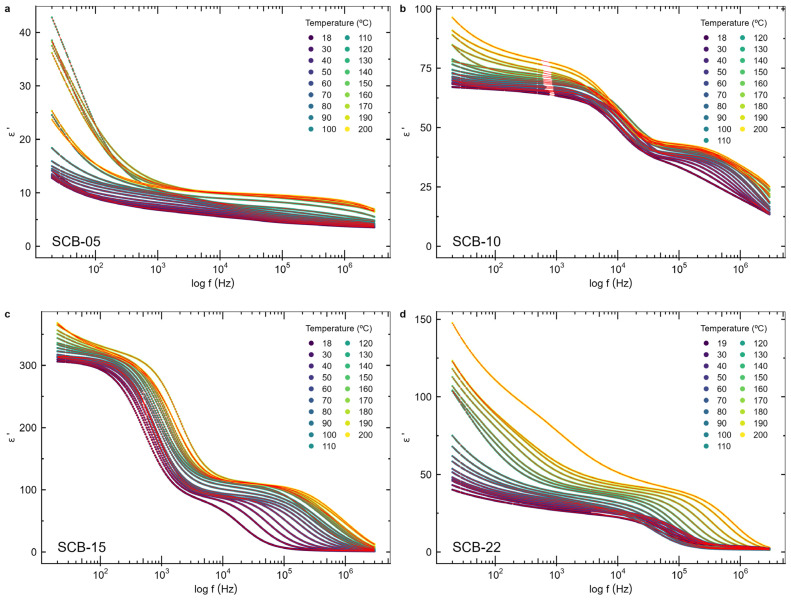
The real component of the electric permittivity of the samples (**a**) SCB-05, (**b**) SCB-10, (**c**) SCB-15, and (**d**) SCB-22 as a function of the frequency. The red lines are fits of the model described in the text.

**Figure 8:**
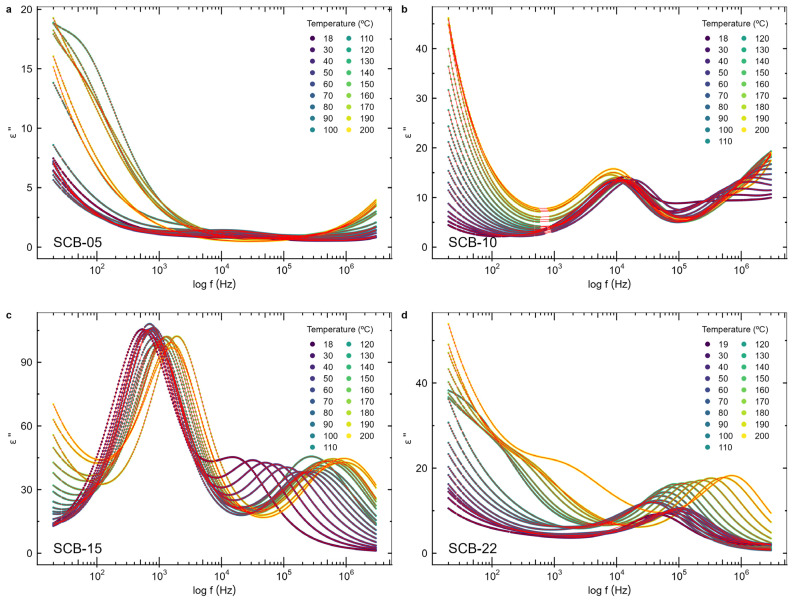
The imaginary component of the electric permittivity of the samples (**a**) SCB-05, (**b**) SCB-10, (**c**) SCB-15, and (**d**) SCB-22 as a function of the frequency. The red lines are fits of the model described in the text.

**Figure 9:**
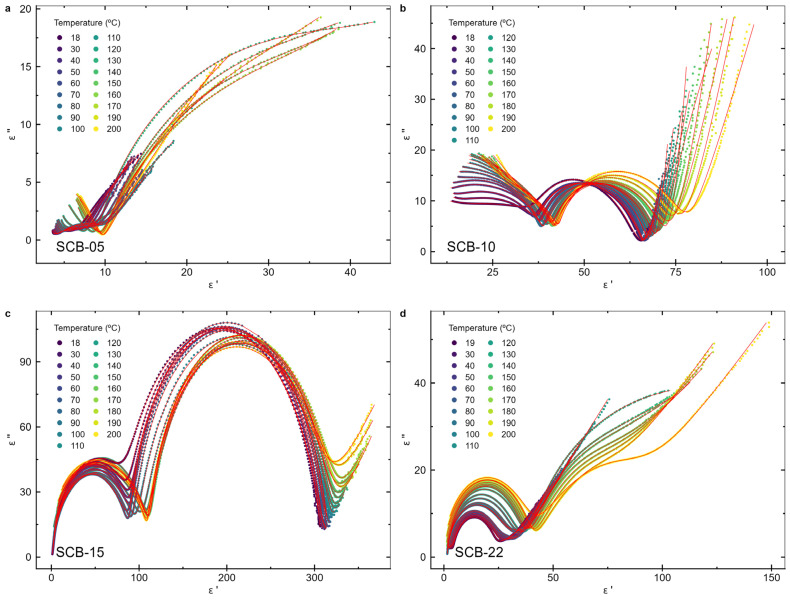
Nyquist plots of the permittivity at temperatures between 18 °C and 200 °C for the samples (**a**) SCB-05, (**b**) SCB-10, (**c**) SCB-15, and (**d**) SCB-22. The red lines are fits of the model described in the text.

Figure 7 and Figure 8 display the frequency-dependent real and imaginary parts of the electric permittivity, respectively, for bilayer samples with varying BaTiO_3_ layer thicknesses, measured between room temperature (18 °C) and 200 °C. Figure 9 shows the Nyquist plot, which illustrates the imaginary versus real parts of the electric permittivity.

Two relaxations are observed, indicated by the two peaks in the imaginary component of the permittivity and the two semicircles in the Nyquist plots. At lower frequencies (<10^2^ Hz), a steep rise in the imaginary component of the electric permittivity is noticeable as the frequency drops (Figure 8), which increases with temperature, suggesting the presence of an electrical conductivity contribution from free charges [13,39], particularly at higher temperatures. Since the bulk conductivity of CoFe_2_O_4_ at room temperature (σ_CFO_ ~ 10^−6^ S/m [40]) is lower than the one for BaTiO_3_ (σ_BTO_ ~ 10^−8^ S/m [41]), this behavior is attributed to the CoFe_2_O_4_ layer. At high frequencies, charge carriers do not have time to respond to the oscillating applied electric field, and the sample capacitance behaves as two insulating capacitors in series, formed by the BTO and CFO layers. In contrast, at low frequencies, the charge carriers in the more conductive CoFe_2_O_4_ layer can follow the oscillating electric field, resulting in the observed strong increase in the permittivity of the films.

The observed relaxations are broad, indicating a distribution of relaxation times and the presence of correlated dipole behavior. Therefore, to fit the electrical permittivity curves, the Havriliak–Negami (HN) model function [27,39,42], with two relaxation times, was used:

(3) $$\varepsilon \left(\omega \right)\;=\;\varepsilon_{\infty }+\sum_{j=1}^{2}\frac{∆\varepsilon_{j}}{{\left[1+{\left(i\omega \tau_{j}\right)}^{\beta_{j}}\right]}^{\gamma_{j}}}$$

where $∆\varepsilon_{j}$ is the intensity associated with each component, $\varepsilon_{\infty }$ is the high-frequency dielectric constant, ω is the angular frequency, $\tau_{j}$ are the relaxation times, and $\beta_{j}$ and $\gamma_{j}$ are parameters that define, respectively, the asymmetry and broadness of the dispersion of each HN function, with the constraints $0<\beta_{j}\leq 1$ and $0<\beta_{j}\gamma_{j}\leq 1$. The Havriliak–Negami model reduces to the Debye function when γ = β = 1.

A conductivity term was introduced to account for the presence of free charges. For purely electronic conduction, the permittivity is imaginary and is given by the equation $\varepsilon \left(\omega \right)=i\frac{\sigma }{\varepsilon_{0}\omega }$ [13,39], where ε_0_ is the vacuum dielectric permittivity, ω is the angular frequency, and σ is the DC conductivity. For ionic charge carriers, which cause electrode or Maxwell–Wagner polarization effects, this equation can be generalized so that the conductivity contribution can be described by the equation $\varepsilon_{cond}\left(\omega \right)=i\frac{\sigma }{\varepsilon_{0}\omega^{s}}$, where s is an exponent, with s≤1 [39]. As such, this term was added to Equation (3) in order to model the conductivity contribution to the dielectric permittivity of the films.

**Figure 10:**
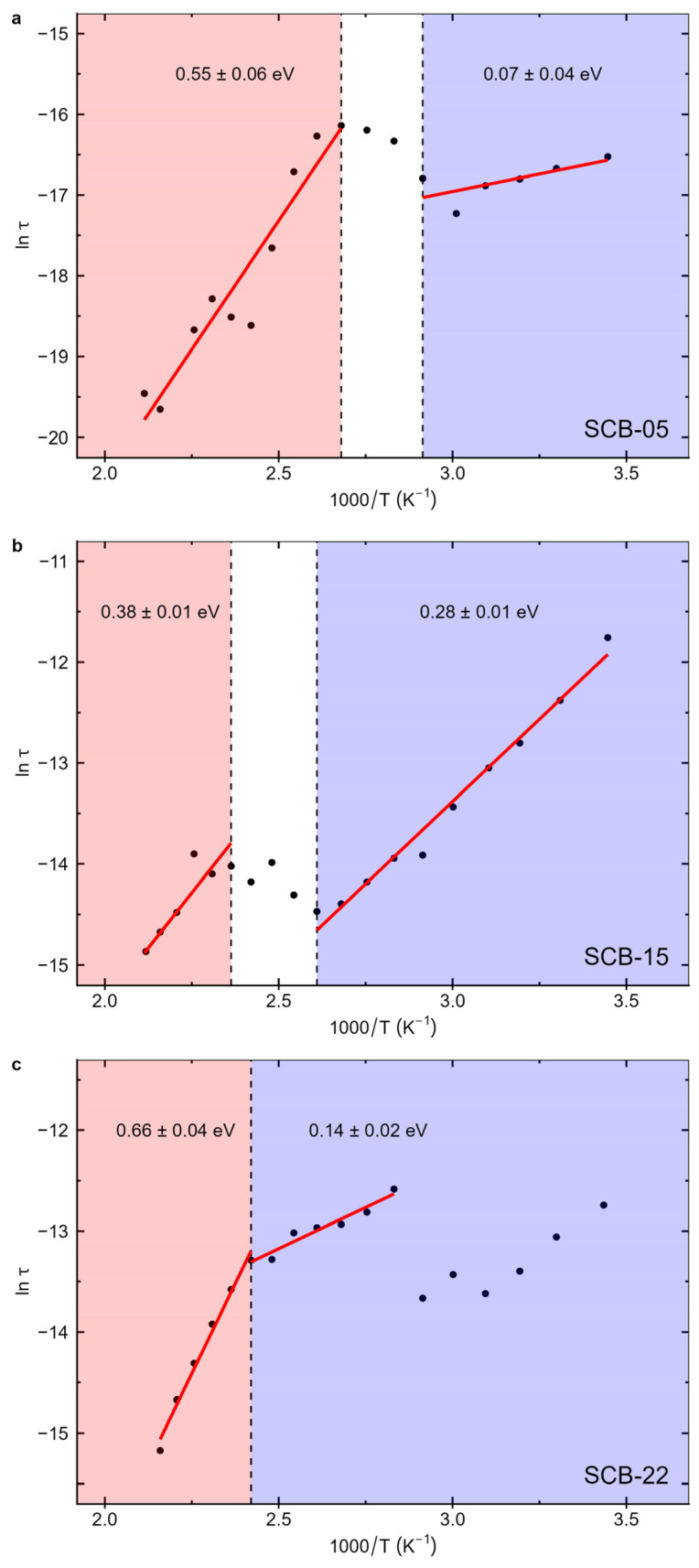
The logarithm of the relaxation times as a function of the inverse of temperature with linear fits for the two different regimes, above and below Tc, for the samples (**a**) SCB-05, (**b**) SCB-15, and (**c**) SCB-22.

The real and imaginary parts of the permittivity were fitted using Equation (3) along with the conductivity contribution, as shown in Figure 7 and Figure 8, respectively, to determine the function parameters for each measured temperature. The corresponding Nyquist plot data with the HN equation fit is presented in Figure 9. The relaxation times (τ) obtained from the HN fit for the main relaxation process present in all samples, occurring in the 10^4^–10^6^ Hz frequency region, exhibit a thermally activated temperature-dependent behavior. Therefore, to determine the activation energies (*E_τ_*), the Arrhenius equation [39] was considered:

(4) $$\tau \left(T\right)=\tau_{0}e^{+\frac{E_{\tau }}{k_{B}T}}$$

where τ_0_ is a constant. Figure 10 shows the logarithm of the relaxation time as a function of the inverse of temperature. Table 4 shows the corresponding activation energies obtained from the relaxation time fits for the bilayer samples.

The graphs in Figure 10 illustrate two distinct regimes: one for temperatures below a characteristic temperature Tc, where the activation energies range from 0.1 to 0.3 eV, and another at higher temperatures, where the activation energies exceed 0.3 eV. The characteristic temperature is in the range of 115 to 130 °C, which is close to the ferroelectric Curie temperature of bulk BaTiO_3_ (Tc ~ 130 °C) [21]. Thus, the observed changes in activation energy reflect the behavior of the BTO layer, which exhibits a paraelectric–ferroelectric phase transition within the measured temperature range.

Regarding the activation energies, oxygen vacancies (*V_O_*) are known to significantly influence the physical properties of BaTiO_3_, such as its conductivity and dielectric relaxation phenomena. Typically, in BaTiO_3_, oxygen vacancies result from the thermal excitations of electrons, inducing the reduction of Ti^4+^ to Ti^3+^ accompanied by the generation of doubly ionized $V_{O}^{**}$ [15]. For a stoichiometric ABO_3_ perovskite, such as BaTiO_3_, the activation energy for oxygen migration is ~2 eV [43,44]. This activation energy decreases with increasing oxygen deficiency: for ABO_2.95_, it is ~1 eV, and for ABO_2.90_, it is ~0.5 eV [44]. However, these energies are consistently higher than those observed here. In fact, in BaTiO_3_, oxygen vacancy migration and associated relaxations tend to occur at higher temperatures, above ~200 °C [43,45,46].

On the other hand, at low temperatures below ~200 °C, it is known that small polarons play an important role in the BaTiO_3_ optical properties [47,48] and charge carrier transport [49]. They involve self-trapped electron polarons mostly localized on the Ti ion sites. The corresponding hopping activation energies are ~0.5 eV or below [50]. These activation energies tend to increase with increasing temperature, changing significantly at Tc, from values ~0.1–0.2 eV around room temperature to ~0.5–0.6 eV above the Curie temperature [50]. These changes are due to the structural transition from a ferroelectric tetragonal phase, which is present at room temperature, to a cubic paraelectric phase, which occurs at high temperatures in BaTiO_3_. This transition induces spontaneous polarization, structure, phonon frequency, and electron–phonon coupling changes. As a result, these alterations in the local environment lead to changes in the activation energies of polarons in BaTiO_3_, as observed in our films.

Regarding the Tc values determined from the dielectric measurements, the transition temperature is significantly below the bulk value for the lowest BaTiO_3_ layer thickness (d_BTO_). Additionally, there is an increase in the transition temperature with increasing d_BTO_, approaching the bulk value for higher BaTiO_3_ layer thicknesses, as shown in Figure 10. In this context, finite-size scaling theory predicts that Tc shifts to lower temperatures compared to the bulk when one or more dimensions of the material are reduced in size, with the shift described by Equation (5) [51]:

(5) $$t=\frac{T_{C\infty }-T_{C}}{T_{C\infty }}={\left(\frac{\xi_{0}}{d_{BTO}}\right)}^{\lambda }$$

where Tc is the film Curie temperature and $T_{C\infty }$ is the Curie temperature of the bulk crystal. The parameter $\xi_{0}$ is the correlation length for the limit T = 0 K and λ is a critical exponent that characterizes the universality class of the material (such as 3D Heisenberg, 3D Ising, etc. [52]). The exponent λ is related to the correlation length (ξ) critical exponent ν ($\xi \propto {\left(T_{C\infty }-T_{C}\right)}^{-\nu }$ [53]) through λ = 1/ν [54,55].

**Figure 11:**
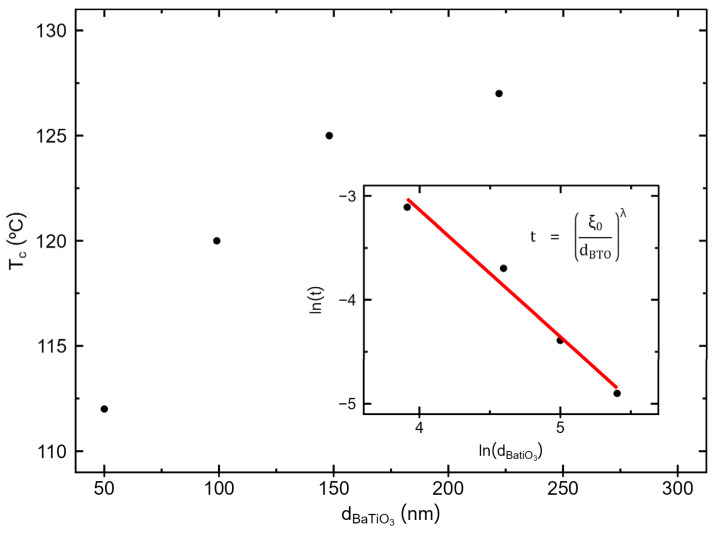
Obtained Curie temperatures as a function of the sample’s barium titanate layer thickness. The insert displays the logarithm of $t=\left(T_{C\infty }-T_{C}\right)/T_{C\infty }$ as a function of the logarithm of the thickness.

According to Equation (5), $\ln\left(t\right)=(\ln\left(\xi_{0}\right)-\ln\left(d_{BTO}\right))/\nu$. Thus, ν and $\xi_{0}$ can be determined from a linear fitting to the graphic of ln(t) versus ln($d_{BTO}$), as shown in the inset of Figure 11. The obtained values are ν = 1/λ = 0.82 ± 0.1 and $\xi_{0}$ = 4.2 ± 0.7 nm. The critical exponent ν is consistent with the 3D random Ising universality class, for which a value of 0.71 has been determined [52]. A similar result was also found in Pb(Zr_0.5_Ti_0.5_)O_3_ thin films [51]. As the critical exponent points to the 3D random Ising universality class rather than the “pure” 3D Ising model (where = 0.627 [52]), this indicates some degree of local disorder and inhomogeneities in the thin films. This can be explained in terms of the formation of a composite structure typically observed within nanoscopic BaTiO_3_ grains, such as those in these films, where they consist of a tetragonal core, a gradient lattice-strained layer, and a surface cubic (non-ferroelectric) layer [6,20,56]. In nanoscopic grains, this surface paraelectric layer becomes more important relative to the grain core contribution. Combined with the presence of grain boundaries that tend to be non-ferroelectric and strongly stoichiometry sensitive in BaTiO_3_ and the presence of the interfacial induced strain due to the CoFe_2_O_4_ layer, particularly in the thinner BaTiO_3_ layer samples. this contributes to disordered grains surface layer, to the Tc reduction and to the observed 3D-random-Ising universality class in our films. 

**Figure 12:**
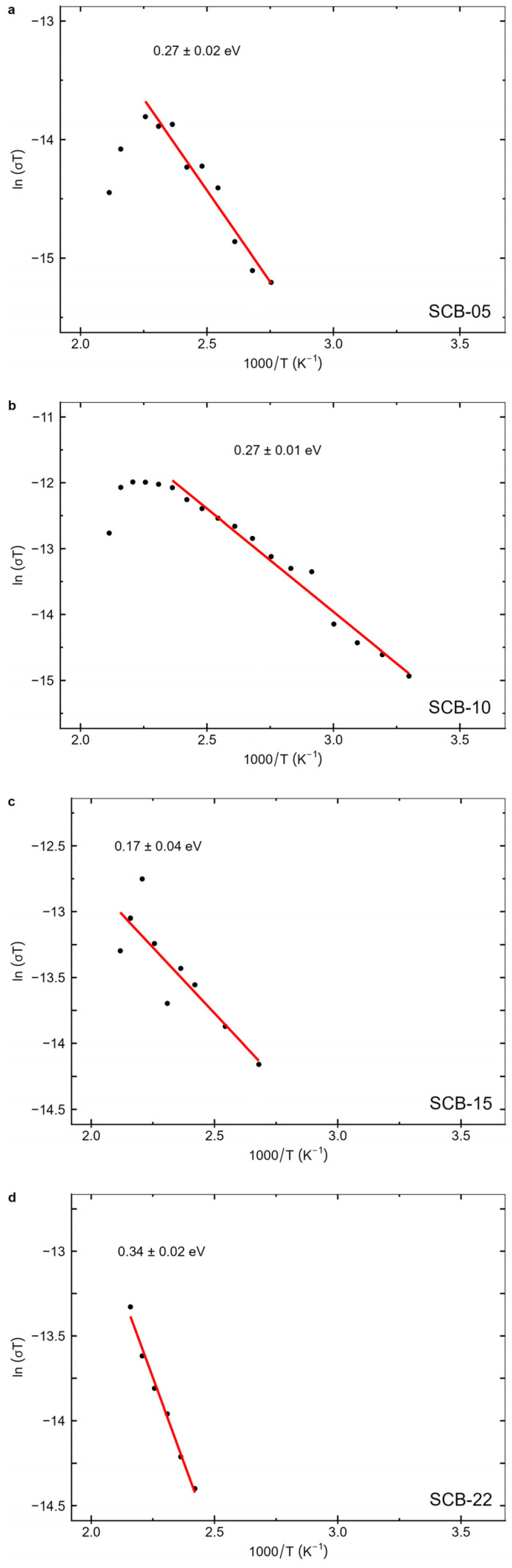
The logarithm of the conductivity time temperatures as a function of the inverse of temperature, with the corresponding linear fits, for the samples (**a**) SCB-05, (**b**) SCB-10, (**c**) SCB-15, and (**d**) SCB-22.

Thermally activated behavior was also found for the temperature dependence of the electric conductivity, determined from the fits with the HN model plus conductivity. This dependence can be described by Equation (6) [57]:

(6) $$\sigma \left(T\right)=\frac{\sigma_{0}}{T}\tau_{0}e^{-\frac{E_{\sigma }}{k_{B}T}}$$

where *σ_0_* is a constant prefactor and *E_σ_* is the activation energy associated with the DC conductivity. Figure 12 shows the logarithm of the DC conductivity times’ temperature as a function of the inverse temperature, obtained for the temperatures where a strong low-frequency conductivity-induced increase in permittivity was observed (Figure 8). Table 4 presents activation energies obtained for the conductivity.

From Figure 12, it can be observed that the fitted Arrhenius behavior tends to span the entire temperature range considered. This is in contrast to what was observed in the relaxation times of the samples, where changes associated with the ferroelectric–paraelectric transition of BaTiO_3_ altered the relaxation time behavior near the Curie temperature. This indicates that the same process is present throughout the entire measured temperature range, suggesting that the conductivity behavior mainly reflects the behavior of CoFe_2_O_4_.

At room temperature, the electric transport and polarization behavior in bulk ferrite are related and arise from the electron hopping between Fe^2+^ and Fe^3+^ ions and hole hopping between Co^3+^ and Co^2+^ ions in the octahedral B sites [58,59,60]. In this context, the distances between cations in tetrahedral (A) sites and between cations in octahedral (B) sites of CoFe_2_O_4_ are typically 0.35 nm and 0.3 nm [61], respectively. Therefore, the activation energy associated with the hopping of electrons and holes between tetrahedral sites is greater than that of the octahedral sites [62]. For this reason, at low temperatures, conductivity is dominated by hopping between the octahedral sites, with an activation energy of the order of 0.5 eV [62]. On the other hand, with increasing temperature, lattice vibrations increase, leading to the existence of another conductivity regime in ferrites, occurring at temperatures above ~500 K. This regime originates by hopping between ions located in tetrahedral sites, with activation energies of the order of 0.8 eV [62]. Given that the obtained values of the activation energy are around 0.3 eV, this suggests that conductivity in the ferrite layer is dominated by hopping between the ions located in the octahedral sites of the cobalt ferrite phase in the films. It may also be related to the onset of oxygen vacancy movement, which has activation energies of ~0.4 eV in the ferrite [58], although this would tend to occur at temperatures higher than those reported here. In this regard, future work will need to address the role of CFO layer structure, chemistry, thickness, and interfacial strain on the dielectric properties, magnetoelectric interactions, and critical temperatures of these bilayer multiferroic thin films.

## 4. Conclusions

In this study, bilayer composite thin films comprising BaTiO_3_ (BTO, piezoelectric)—CoFe_2_O_4_ (CFO, magnetostrictive) layers on highly doped conductive Si (001) substrates, compatible with current Si electronics, were successfully fabricated using laser ablation. A comprehensive characterization of their structural, microstructural, spectroscopic, non-linear optical, magnetic, electric, and dielectric properties was conducted. X-ray diffraction, Raman spectroscopy, and non-linear optical analysis confirmed the tetragonal ferroelectric structure of the BTO layer and the cubic spinel phase of the cobalt ferrite layer. Notably, a strong interfacial interaction at low BTO thicknesses was observed, resulting in compressive strain on the CFO layer, enhanced BTO layer tetragonality (higher c/a), and wasp-waisted magnetic hysteresis cycles. The electrical permittivity studies, supported by modeling and fitting, revealed crucial insights into activation energies, conductivity behaviors, ferroelectric Curie temperature variations, and characteristic frequencies. The BTO layer exhibited temperature-dependent relaxation time behavior attributed to small-polaron transport, with a significant shift at the BTO Curie temperature. Furthermore, the thickness-dependent Curie temperature behavior of BTO displayed critical exponents associated with the 3D random Ising universality class, which were attributed to the composite structure of BTO grains in the films, consisting of a bulk-like inner structure, a gradient-strained layer, and surface disorder. DC conductivity analysis indicated that the CFO layer’s conductivity mechanism primarily involves hopping between ions located at octahedral sites of the ferrite. These findings not only illuminate the complex interplay between structural and functional properties in multiferroic bilayer systems but also pave the way for tailoring their characteristics for potential applications in next-generation multifunctional fast spintronic devices.

## Figures and Tables

**Table 1:** Pulsed laser-ablation deposition conditions used for each layer. Variables abbreviated as follows: O_2_ atmosphere pressure (P); pulse frequency (f); target-substrate distance (d); and substrate temperature (T).

Material	P (mbar)	f (Hz)	d (cm)	T (°C)
CoFe_2_O_4_	0.1	10	3.5	650
BaTiO_3_	0.03	5	5.0	700

**Table 2:** The thickness of the layers, determined from the scanning electron micrographs of the sample cross-sections in Figure 1; lattice parameters of the CoFe_2_O_4_ layer in the samples (for samples SCB-5 and SCB-10, the values are from the grazing incidence measurements) and grain sizes of the CoFe_2_O_4_ and BaTiO_3_ layers.

Sample	Thickness CoFe_2_O_4_ (nm)	Thickness BaTiO_3_ (nm)	Latt. Param. CoFe_2_O_4_ a (Å)	Grain Size CoFe_2_O_4_ (nm)	Grain Size BaTiO_3_ (nm)
SCB-05	66	50	8.324	12	38
SCB-10	72	99	8.357	28	36
SCB-15	79	148	8.360	35	38
SCB-22	72	222	8.322	28	43

**Table 3:** Atomic percentages obtained by EDX and their ratios.

Sample	Co (%)	Fe (%)	Ba (%)	Ti (%)	Co/Fe	Ba/Ti
SCB-10	17.78	30.83	26.94	24.44	0.58	1.10
SCB-15	14.85	23.51	30.93	30.72	0.63	1.01
SCB-22	7.83	12.78	44.25	35.15	0.61	1.26

**Table 4:** Activation energies obtained from the relaxation times (E_τ_) and conductivity fits. T_C_ is the characteristic temperature at which there is a change in the relaxation time’s behavior.

Sample	E_τ_ LT (eV)	E_τ_ HT (eV)	E_σ_ (eV)	T_C_ (°C)
SCB-05	0.07	0.55	0.27	112
SCB-10			0.27	120
SCB-15	0.29	0.38	0.24	120
SCB-22	0.14	0.65	0.33	130

## Data Availability

The original contributions presented in the study are included in the article, further inquiries can be directed to the corresponding authors.
